# Coping, Perceived Stress, And Life Satisfaction Among Female Doctors in a Low Middle-Income Country

**DOI:** 10.1192/j.eurpsy.2025.629

**Published:** 2025-08-26

**Authors:** S. Q. Bokhari, A. Butt, A. Manzoor, A. Waheed, S. Ijaz

**Affiliations:** 1 Psychiatry, Services Institute of Medical Sciences; 2 Psychology, Services hospital Lahore, Lahore, Pakistan

## Abstract

**Introduction:**

This research explores the intricate relationship between coping strategies, perceived stress levels, and life satisfaction among female medical professionals. The medical field is known for its rigorous demands, and understanding how lady doctors manage stress and its impact on their overall life satisfaction is crucial. While previous studies have shed light on stress-related issues in medical undergraduates, there is a significant gap in research focused on the well-being of practicing female doctors.

**Objectives:**

The objectives of this study are to investigate the relationship between coping behaviors and stress levels among lady doctors, assess the role of coping behaviors in shaping life satisfaction, explore the connections between coping behaviors, life satisfaction, and stress, and analyze the influence of demographic factors such as age and marital status on coping life satisfaction, and stress perception.

**Methods:**

This study utilizes a quantitative research design and a purposive sample of lady doctors from government hospitals in Pakistan. Key measures include the COPE Inventory to assess coping behaviors, the Satisfaction with Life Scale to gauge life satisfaction, and the Perceived Stress Scale to measure stress levels. These tools allow for a comprehensive examination of the intricate interplay between these variables. SPSS 21 was used to analyze the data.

**Results:**

Results indicated that Coping is negatively correlated with Stress (r = -.29, n = 100, p = 0.05) meaning that higher coping strategies are associated with lower stress levels. Similarly, Coping is positively correlated with Life Satisfaction (r = .36, n = 100, p = 0.05) indicating that higher coping strategies are associated with higher life satisfaction. Likewise, Stress is negatively correlated with Life Satisfaction (r = -.22, n = 100, p = 0.05), suggesting that higher stress levels are associated with lower life satisfaction. Also, there is a statistically significant difference in coping between Single and Married individuals (t = 2.2, df = 36.6, p = 0.03), with Single individuals showing higher coping scores.

**Conclusions:**

The findings of this study provide valuable insights into the psychological well-being of female medical professionals in Pakistan. This research contributes to the broader discourse on the well-being of healthcare professionals, shedding light on the unique experiences of female doctors in a challenging healthcare environment. Ultimately, it aims to inform policies and practices that support the psychological resilience and job satisfaction of female doctors, ensuring they can continue providing high-quality healthcare services to their communities.

**Disclosure of Interest:**

None Declared

